# The association between reduced kidney function and hearing loss: a cross-sectional study

**DOI:** 10.1186/s12882-020-01810-z

**Published:** 2020-04-22

**Authors:** Wenwen Liu, Qinqin Meng, Yafeng Wang, Chao Yang, Lili Liu, Huaiyu Wang, Zaiming Su, Guilan Kong, Yaohui Zhao, Luxia Zhang

**Affiliations:** 1 Department of Epidemiology and Biostatistics, School of Public Health, Peking University, Beijing, China; 2 Institute of Social Science Survey, Peking University, Beijing, China; 3 Renal Division, Department of Medicine, Peking University First Hospital, Peking University Institute of Nephrology, 8 Xishiku Street, Xicheng District, Beijing, 100034 China; 4 National Institute of Health Data Science, Peking University, 38 Xueyuan Road, Haidian District, Beijing, 100191 China; 5 Center for Data Science in Health and Medicine, Peking University, Beijing, China; 6 National School of Development, Peking University, 5 Yiheyuan Road, Haidian District, Beijing, 100871 China

**Keywords:** CHARLS, Reduced kidney function, eGFR, Hearing loss, Multivariable logistic regression

## Abstract

**Background:**

The relationship between kidney function and hearing loss has long been recognized, but evidence of this association mostly comes from small observational studies or other populations. The aim of this study is to explore the association between reduced kidney function and hearing loss in a large population-based study among the middle-aged and elderly Chinese.

**Methods:**

Data collected from the Chinese Health and Retirement Longitudinal Study (CHARLS) in 2015 were used for analysis. A cross-sectional study was conducted among 12,508 participants aged 45 years and older. Hearing loss, the outcome of this study, was defined according to interviewees’ responses to three survey questions related to hearing in the CHARLS. Estimated glomerular filtration rate (eGFR) was employed to assess kidney function, and participants were classified into three categories based on eGFR: ≥90, 60–89 and < 60 mL/min/1.73 m^2^. Multivariable logistic regression was employed to adjust for potential confounders, including demographics, health-related behaviors, and cardiovascular risk factors.

**Results:**

The overall prevalence of self-reported hearing loss in the study population was 23.6%. Compared with participants with eGFR ≥90 mL/min/1.73 m^2^, participants with eGFR of 60–89 mL/min/1.73 m^2^ (odds ratio [OR]: 1.11, 95% confidence interval [CI]: 1.00–1.23) and eGFR < 60 mL/min/1.73 m^2^ (OR: 1.25, 95% CI: 1.04–1.49) showed increased risk of hearing loss after adjusting for potential confounders.

**Conclusions:**

Reduced kidney function is independently associated with hearing loss. Testing for hearing should be included in the integrated management of patients with chronic kidney disease.

## Background

The World Health Organization (WHO) reported that approximately 432 million adults suffered from disabling hearing loss in 2018 and estimated that over 900 million people will have disabling hearing loss by 2050 [1]. Hearing loss in adults not only brings about communication difficulties in daily life but also has negative effects on an individual’s cognitive and psychosocial function. Hearing loss could lead to social isolation, financial strain, and a low health-related quality of life [2–4]. Because most cases of hearing loss are acquired and difficult to recover from, but preventable, exploring the risk factors of hearing loss is of great significance.

The effects of aging on the auditory system are considered the leading cause of adult-onset hearing loss [5]. The association between hearing loss and genetic mutations [6], noise exposure [7], use of ototoxic drugs in treatment [8, 9], and chronic diseases such as hypertension and diabetes [10, 11] has also been demonstrated in existent studies.

Kidney disease has become a public health issue of global concern in recent years. Since the first report on Alport syndrome revealed that hereditary familial nephritis is related to sensorineural deafness in 1927 [12], several other congenital syndromes such as Fabry disease, branchio-oto-renal syndrome, Alstrom syndrome, and Bartter syndrome, have been recognized to have both hearing and kidney manifestations [13–15]. Some observational studies have assessed the auditory function of patients with end-stage kidney disease (ESKD) or receiving kidney replacement therapy [16–19]. For example, Meena et al. [18] studied 50 cases of ESKD and 50 healthy volunteers and found that 28% of the former but only 6% of the latter have sensorineural hearing loss. Zeigelboim et al. [17] found that patients with ESKD have significantly higher hearing thresholds for high frequencies than the control group. Here the hearing threshold refers to the minimum level of sound that evokes an auditory sensation [20]. Renda et al. [19] also found a significant association between the duration of hemodialysis and hearing loss in children aged 6–18 years with dialytic chronic kidney disease (CKD).

The kidney and cochlea have common antigenicity and similar physiologic mechanisms involving the transport of fluid and electrolytes, which might explain the hearing loss in patients with kidney disease [21]. Some possible aetiological factors related to hearing loss in kidney failure patients have also been reported, including electrolyte disturbances, hypertension, the use of ototoxic drug and hemodialysis treatment [21–23].

Unfortunately, most of existing studies have small sample sizes or enrolled patients with kidney failure; moreover, few studies have focused on the association between hearing loss and mildly-to-moderately reduced kidney function in a large population [24, 25]. Vilayur et al. [24] presented the first community-based study on an Australian population and demonstrated an association between moderate CKD and hearing loss. Seo et al. [25] used the Korean National Health and Nutritional Examination Survey and found that individuals with CKD are more likely to have hearing impairment than those without. But the sample size of their study was also relatively small and only the hearing status of two eGFR groups, i.e., ≥60 and < 60 mL/min/1.73 m^2^ were compared. However, Gupta et al. [26] found that there is no significant association between GFR estimated using the serum creatinine-based equation and risk of incident hearing loss.

The prevalence of hearing loss is consistently higher in middle-aged and older populations than in younger cases, but evidence of the association between reduced kidney function and hearing loss in the Chinese middle-aged and older population is limited. We conducted a cross-sectional population-based study to explore the relationship between reduced kidney function and hearing loss by taking advantage of a representative sample of the general population aged 45 years and older and the strict quality control process of the Chinese Health and Retirement Longitudinal Study (CHARLS) [27].

## Methods

### Study population

The CHARLS, a nationally representative survey of China’s middle-aged and elderly population, provides a high-quality public micro-database with social, economic, and health information. Samples were selected by using multistage probability sampling taking into consideration regional and socioeconomic disparities. The probability-proportional-to-size (PPS) sampling technique was used to select 150 county-level units from all counties of China except Tibet. PPS sampling was also applied to select three primary sampling units (PSUs) from each county-level unit. PSUs represent the lowest level of government organization and consist of administrative villages (*cun*) in rural areas and neighborhoods (*shequ* or *juweihui*) in urban areas. Eighty or more households with age-eligible members were selected within each PSU, and one age-eligible member was randomly selected from qualified households. If the chosen person was willing to participate, this person and his or her spouse were interviewed. All stages of sampling were conducted by a computer to avoid potential biases arising from human manipulation.

CHARLS also has good cross-study comparability of results because the survey instrument was developed on the basis of the best international practices and harmonized with over 25 leading international research studies in the Health and Retirement Study model [27].

The data used in our study were obtained from the CHARLS dataset collected in 2015, which included a total of 20,967 individuals. Information on demographic characteristics, health-related behaviors and lifestyles, and health status were collected through face-to-face interviews using the questionnaire. Most of the interviewers were recruited from local colleges and universities and had received 9 days of rigorous training on the questionnaire content, interview techniques, security and quality control, and face-to-face interview practices. The surveyed data were recorded by using a computer-assisted personal interview (CAPI) system, which could help substantially improve the quality of the surveyed dataset by detecting errors in data inputs. When the interviewer enters an input item with a logical error or abnormal value, the system will pop out a message to alert the interviewer to this dubious entry. In this way, the participants no longer need to read the questionnaire, and the interviewers would ask them face to face and help them understand the questions and corresponding options. Anthropometric and physical measurements were provided, and blood samples were collected by trained nurses from township hospitals or China’s Center for Disease Prevention and Control (CDC) according to the standard protocol.

CHARLS was approved by the Biomedical Ethics Review Committee of Peking University (IRB00001052–11015), and all participants signed informed consent before participation.

**Fig. 1:**
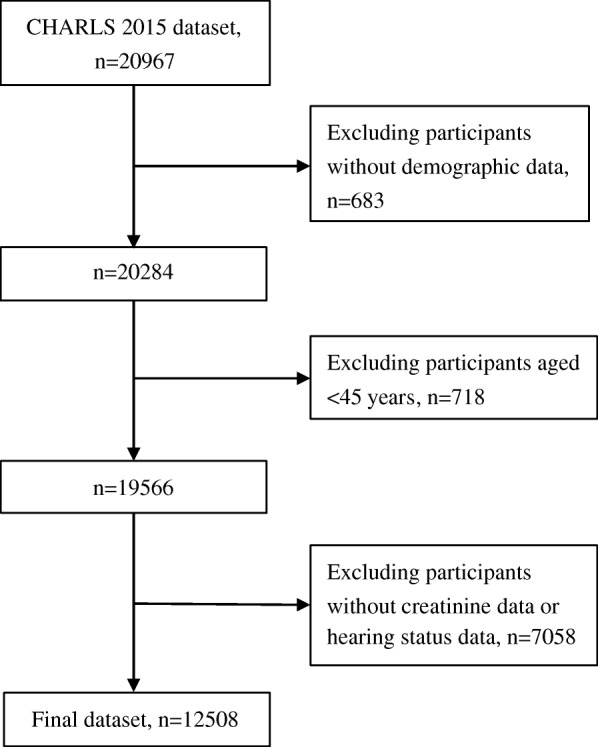
Flow chart of the participant selection

The exclusion criteria of the study were as follows: (1) demographic data were not recorded; (2) aged < 45 years old; and (3) creatinine or hearing status data were not recorded. A total of 12,508 participants were included in our final analysis. The participant selection process is shown in Fig. 1.

### Hearing loss

In our study, hearing loss was identified through self-reporting. Objective measurement, such as audiometry, was not provided in CHARLS, but several studies have demonstrated the reliability of self-reported hearing loss [28, 29]. We used hearing-related questions in the CHARLS survey to determine whether a patient had hearing loss. The hearing-related CHARLS survey questions are listed in Table 1. Participants were asked these questions by trained investigators through face-to-face interviews. A participant was defined as having hearing loss if he or she met one of the following three criteria: 1) had a hearing problem; 2) wore a hearing aid; and 3) had a poor hearing status.

**Table 1:** Hearing related questions in the CHARLS survey

(1) Do you have a hearing problem?	
1. Yes	
2. No	
(2) Do you ever wear a hearing aid?	
1. Yes	
2. No	
(3) Would you say your hearing is excellent, very good, good, fair, or poor? (How is your hearing with a hearing aid if you normally use it? How is your hearing without a hearing aid if you normally don’t use it?)	
1. Excellent	
2. Very good	
3. Good	
4. Fair	
5. Poor	

### Reduced kidney function

The estimated glomerular filtration rate (eGFR) is considered the best overall index of kidney function in health and disease [30]. In this study, we determined eGFR using the Chronic Kidney Disease Epidemiology Collaboration (CKD-EPI) equation [31] as follows:

1 $$ \mathrm{GFR}\ \left(\mathrm{mL}/\min /1.73\ {\mathrm{m}}^2\right)=\mathbf{141}\times \min {\left(\mathrm{Scr}/\upkappa, \mathbf{1}\right)}^{\upalpha}\times \max {\left(\mathrm{Scr}/\upkappa, \mathbf{1}\right)}^{-\mathbf{1.209}}\times {\mathbf{0.993}}^{\mathrm{Age}}\times \mathbf{1}.\mathbf{018}\ \left[ if\ female\right]\times \mathbf{1}.\mathbf{159}\ \left[ if\ black\right] $$

where Scr represents serum creatinine measured in units of mg/dL. κ is 0.7 for females and 0.9 for males, α is − 0.329 for females and − 0.411 for males, min refers to the minimum value of Scr/κ and 1, and max refers to the maximum value of Scr/κ and 1.

At the beginning of this study, we attempted to classify the eGFR into five groups: ≥90, 60–89, 30–59, 15–29, and < 15 mL/min/1.73 m^2^, according to the different stages of CKD defined by the Kidney Disease: Improving Global Outcomes (KDIGO) guideline [32]. We calculated the numbers and percentages of participants in different eGFR groups, and found that only 74 (0.6%) participants with eGFR of 15–29 mL/min/1.73 m^2^ and 8 (0.1%) participants with eGFR < 15 mL/min/1.73 m^2^, which may lead to limited statistical power to examine the association between eGFR and hearing loss. This is a study based on general population, and thus there are less people in lower eGFR groups. Therefore, we finally used only three eGFR groups of ≥90, 60–89, and < 60 mL/min/1.73 m^2^ for analysis, which represented normal kidney function, mildly reduced kidney function, and moderately to severely reduced kidney function, respectively.

### Other predictor variables

We chose predictor variables by referring to hearing loss risk factors reported in the existent literatures [5, 33] and their availabilities in the CHARLS dataset. Besides eGFR, predictor variables used in the regression models included demographic characteristics (i.e., age, gender, education, area of residence), health-related behaviors (i.e., smoking and drinking status) and cardiovascular risk factors (i.e., body mass index [BMI], central obesity, hypertension, diabetes, stroke, high-density lipoprotein [HDL] cholesterol, and low-density lipoprotein [LDL] cholesterol). Demographics and health-related behaviors data were obtained from the questionnaire. BMI was defined as weight in kilograms divided by the square of height in meters, and acquired through physical measurements. Participants were categorized as underweight (< 18.5 kg/m^2^), normal weight (18.5 to 24.9 kg/m^2^), overweight (25.0 to 29.9 kg/m^2^), and obese (≥30 kg/m^2^) according to their BMI. In our study, central obesity was defined as a waist circumference of ≥80 cm for females and ≥ 102 cm for males. Hypertension was defined as mean systolic blood pressure (SBP) ≥140 mmHg, mean diastolic blood pressure (DBP) ≥90 mmHg, or a self-report of hypertension. Diabetes was defined as fasting plasma glucose ≥126 mg/dL, HbA1c concentration ≥ 6.5%, or a self-reported doctor diagnosis. Stroke was defined as a self-reported history of doctor diagnosis. HDL and LDL cholesterol levels were directly obtained from the results of laboratory tests.

### Statistical analysis

Descriptive statistics for continuous variables are presented by using means and standard deviations (SD), while frequencies and percentages are used to describe categorical variable characteristics. Student’s *t*-test was used to compare the mean values of continuous variables between participants with and without hearing loss, and differences in hearing loss prevalence among different categorical variable groups were tested by using Pearson’s *χ*^2^ test. The association between reduced eGFR and hearing loss was modeled by using a logistic regression function, and odds ratios (ORs) with 95% confidence intervals (CIs) of hearing loss for different eGFR categories were calculated. Multivariable logistic regression models were constructed to adjust for potential confounding variables (i.e., age [45–54 years, 55–64 years, ≥65 years], gender [male, female], education [illiterate, literate, primary school, middle school, high school and above], area of residence [urban or rural], smoking [never, current, past], drinking [never, current, past], BMI [underweight, normal weight, overweight, obese], central obesity [yes or no], hypertension [yes or no], diabetes [yes or no], stroke [yes or no], HDL cholesterol [continuous value], and LDL cholesterol [continuous value]). To build the multivariable logistic regression models, we used a separate unknown category to represent missing data for BMI, central obesity, and stroke, which had missing rates greater than 1%. Other variables included in the models that had missing rates lower than 1% were not preprocessed as cases containing missing data were automatically deleted during logistic regression analysis.

All analyses were performed by using STATA software (version 14.0). All *p* values were based on two-sided tests with a significance level of 0.05.

## Results

A total of 12,508 participants were included in the final dataset for analysis. The characteristics of participants are described in Table 2. The mean age was 60.5 years, and 52.9% of the participants were females. Significant differences in hearing loss prevalence were observed among participants of different age, educational background, area of residence, smoking status, drinking status, BMI, chronic health status (i.e., with/without hypertension, with/without diabetes, with/without stroke), SBP, DBP, and eGFR (all *p* values < 0.001). The distribution of self-reported hearing loss prevalence is presented in Table 3. Overall, the prevalence of self-reported hearing loss in our study was 23.6, and 35.8% of the participants with eGFR < 60 mL/min/1.73 m^2^ reported hearing loss. The prevalence of hearing loss in this group was nearly twice that in the group with eGFR ≥90 mL/min/1.73 m^2^ (19.4%).

**Table 2:** Characteristics of the participants

Variables	Overall (***n*** = 12,508)	Hearing loss (***n*** = 2946)	No hearing loss (***n*** = 9562)	***P*** value
**Age (years)**	60.5 ± 9.6	64.8 ± 10.1	59.1 ± 9.1	< 0.001
**Gender**				0.832
Male	5889 (47.1)	1382 (46.9)	4507 (47.1)	
Female	6619 (52.9)	1564 (53.1)	5055 (52.9)	
**Education**				< 0.001
Illiterate	3851 (30.8)	1249 (42.4)	2602 (27.3)	
Literate	947 (7.6)	237 (8.0)	710 (7.4)	
Primary	3232 (25.9)	788 (26.8)	2444 (25.6)	
Middle	3016 (24.1)	500 (17.0)	2516 (26.4)	
High and above	1449 (11.6)	172 (5.8)	1277 (13.4)	
**Rural**	10,191 (81.7)	2523 (85.7)	7668 (80.4)	< 0.001
**Smoking**				< 0.001
Never	7388 (59.1)	1702 (57.8)	5686 (59.5)	
Current	3475 (27.8)	768 (26.1)	2707 (28.3)	
Past	1638 (13.1)	474 (16.1)	1164 (12.2)	
**Drinking**				< 0.001
Never	6717 (53.8)	1611 (54.8)	5106 (53.5)	
Current	4381 (35.1)	912 (31.0)	3469 (36.3)	
Past	1392 (11.1)	419 (14.2)	973 (10.2)	
**BMI (kg/m**^**2**^**)**				< 0.001
< 18.5	702 (5.6)	233 (7.9)	469 (4.9)	
18.5–24.9	7137 (57.1)	1730 (58.7)	5407 (56.6)	
25.0–29.9	3717 (29.7)	758 (25.7)	2959 (31.0)	
≥ 30.0	688 (5.5)	155 (5.3)	533 (5.6)	
**Central Obesity**	3307 (26.4)	753 (25.6)	2554 (26.7)	0.383
**Hypertension**	5584 (44.7)	1543 (52.5)	4041 (42.3)	< 0.001
**Diabetes**	2286 (18.3)	638 (21.7)	1648 (17.3)	< 0.001
**Stroke**	329 (2.6)	116 (3.9)	213 (2.2)	< 0.001
**HDL Cholesterol (mg/dL)**	51.2 ± 11.6	51.4 ± 12.1	51.2 ± 11.5	0.424
**LDL Cholesterol (mg/dL)**	102.5 ± 29.0	101.8 ± 29.2	102.7 ± 29.0	0.152
**SBP (mmHg)**	128.7 ± 20.0	131.2 ± 21.1	128.0 ± 19.6	< 0.001
**DBP (mmHg)**	75.8 ± 11.7	75.2 ± 11.5	75.9 ± 11.7	0.006
**eGFR (mL/min/1.73 m**^**2**^**)**	90.1 ± 16.4	86.0 ± 17.1	91.3 ± 16.0	< 0.001
**eGFR groups (mL/min/1.73 m**^**2**^**)**				< 0.001
≥ 90	7498 (60.0)	1456 (49.4)	6042 (63.2)	
60–89	4315 (34.5)	1241 (42.1)	3074 (32.2)	
< 60	695 (5.6)	249 (8.5)	446 (4.7)	

Note: Data are expressed as number (%) or mean ± SD, unless otherwise indicated

†Number (%) of missing data points: 13 (0.10%) in education, 30 (0.24%) in area of residence, 7 (0.06%) in smoking, 18 (0.14%) in drinking, 264 (2.11%) in BMI, 443 (3.54%) in central obesity, 25 (0.20%) in hypertension, 38 (0.30%) in diabetes, 146 (1.17%) in stroke, 1 (0.00%) in LDL cholesterol, 259 (2.07%) in SBP, 259 (2.07%) in DBP

Abbreviations: *BMI* body mass index, *HDL* high-density lipoprotein, *LDL* low-density lipoprotein, *SBP* systolic blood pressure, *DBP* diastolic blood pressure, *eGFR* estimated glomerular filtration rate

**Table 3:** Prevalence of hearing loss in different age and eGFR groups

	Individuals	Hearing loss	Prevalence (%)
**Total**	12,508	2946	23.6
**Age groups (years)**			
45–54	3935	534	13.6
55–64	4498	902	20.1
≥ 65	4075	1510	37.1
**eGFR groups (mL/min/1.73 m**^**2**^**)**			
≥ 90	7498	1456	19.4
60–89	4315	1241	28.8
< 60	695	249	35.8

The results of logistic regression analysis of the association between eGFR and hearing loss after adjusting for potential confounders are listed in Table 4. Compared with participants with eGFR ≥90 mL/min/1.73 m^2^, the ORs of participants with eGFR of 60–89 mL/min/1.73 m^2^ and eGFR < 60 mL/min/1.73 m^2^ were 1.11 (95%CI, 1.00–1.23; *p* = 0.043) and 1.25 (95%CI, 1.04–1.49; *p* = 0.017), respectively. The detailed multivariable logistic regression analysis results are shown in Table S1 of the Additional file.

**Table 4:** Odds ratios (ORs) and 95% confidence intervals (CIs) for hearing loss in relation to the eGFR categories

Variable	Model 1		Model 2		Model 3		Model 4	
	OR (95%CI)	***p***-value	OR (95%CI)	***p***-value	OR (95%CI)	***p***-value	OR (95%CI)	***p***-value
**eGFR (mL/min/1.73 m**^**2**^**)**								
≥ 90	Reference		Reference		Reference		Reference	
60–89	1.68 (1.54–1.83)	< 0.001	1.08 (0.98–1.19)	0.122	1.11 (1.00–1.22)	0.043	1.11 (1.00–1.23)	0.043
< 60	2.32 (1.96–2.73)	< 0.001	1.30 (1.09–1.55)	0.004	1.29 (1.08–1.54)	0.006	1.25 (1.04–1.49)	0.017

Model 1: Unadjusted;

Model 2: Adjusted for age;

Model 3: Adjusted for age, gender, education, area of residence, smoking, and drinking;

Model 4: Adjusted for age, gender, education, area of residence, smoking, drinking, BMI, central obesity, hypertension, diabetes, stroke, HDL cholesterol, and LDL cholesterol

## Discussion

The results of our study indicated that 23.6% of middle-aged and older Chinese have hearing loss and that this prevalence was higher at older ages. In the process of exploring the association between eGFR and hearing loss, we observed that the odds of hearing loss are higher among participants with lower eGFR.

Comparing the prevalence of hearing loss observed in this study with previous studies is difficult because different studies included participants belonging to different age groups and defined and measured hearing loss using different instruments. Pure-tone audiometry and self-reporting are two common ways to assess hearing status in hearing-related studies [24, 25, 33–38]. Pure tone audiometry detects the hearing threshold at a specific frequency [20]. Most studies [24, 33] performed pure-tone audiometry at 0.5, 1, 2, and 4 kHz. According to the WHO’s recommendations [39], an average threshold of > 25 dB hearing level (HL) in the better-hearing ear is defined as hearing loss and an average threshold of > 40 dB HL in the better-hearing ear is defined as disabling hearing loss. Two of the most recent large-scale hearing loss studies [33, 40] based on the Chinese population were published nearly a decade apart. In 2006, Sun et al. [40] reported that 11.0% of older adults (≥60 years) could be diagnosed as hearing disabled (> 40 dB HL); this study was based on the data of the Second China National Sample Survey on Disability. In 2015, Gong et al. [33] conducted a survey including 6984 older adults (≥60 years) in the four provinces of Jilin, Guangdong, Gansu, and Shaanxi of China and reported prevalences of hearing loss (> 25 dB HL) and disabling hearing loss (> 40 dB HL) of 58.9 and 24.1% respectively. A study [34] based on the Health Survey for England 2014, a nationally representative cross-sectional survey, reported that 26% of men and 20% of women aged 45 years and older have hearing loss (> 35 dB HL at 3.0 kHz of the better-hearing ear). Amieva et al. [36] used self-perceived hearing loss in their study and conducted a short questionnaire survey to assess hearing loss in elderly participants (≥65 years) randomly selected from the general French population. In this study, 4% of the participants reported major hearing loss and 31% reported moderate hearing loss. A WHO report [1] showed that approximately one-third of the population aged over 65 years worldwide suffer from disabling hearing loss. In the present study, the prevalences of hearing loss among participants aged 45–54, 55–64, and ≥ 65 years were 13.6, 20.1, 37.1%, respectively, which is similar to reports from England, France and the WHO.

The association between reduced kidney function and hearing loss found in our study is consistent with the results of some previously published studies [24, 25]. Seo et al. [25] found that eGFR < 60 mL/min/1.73 m^2^ has a significant independent influence on the hearing status of adults. The authors also reported that the OR of hearing impairment in participants with eGFR < 60 mL/min/1.73 m^2^ compared with those having eGFR ≥60 mL/min/1.73 m^2^ is 1.25 (95%CI, 1.12–1.64), after adjusting for age, sex, smoking, alcohol, BMI, diabetes mellitus, hypertension, dyslipidemia and microalbuminuria. Similarly, Vilayur et al. [24] defined moderate CKD as eGFR < 60 mL/min/1.73 m^2^ and found an independent association between moderate CKD and hearing loss with an OR of 1.43 (95%CI, 1.10–1.84). In a prospective study of 1843 individuals, Gupta et al. [26] estimated GFR by using an equation that considers SCr and cystatin C and found that lower eGFR is significantly associated with incident hearing loss at speech frequencies but not at high frequencies. However, when they estimated GFR by using an SCr-based equation, no significant association could be found between either lower baseline eGFR or decline in eGFR and incident hearing loss. This result is inconsistent with our finding, and the difference observed may be due to differences in study populations, definitions of hearing loss, and SCr measurement. Our study provided valid epidemiological evidence of the association between reduced kidney function and hearing loss in middle-aged and older Chinese population, and is based on a nationally representative large-scale dataset of high quality. We classified eGFR into three groups and found that participants in two eGFR groups, specifically, those with eGFR of 60–89 and < 60 mL/min/1.73 m^2^ have higher odds of hearing loss, compared with participants with eGFR ≥90 mL/min/1.73 m^2^. These results suggest that, not only the CKD patients at stage 3–5, but also those at stage 2 have an increased risk of hearing loss. More attention should be paid to the hearing status of CKD patients from early stage to enable the implementation of suitable interventions at an appropriate time and reduce the burden of the disease.

Possible mechanisms of the association between reduced kidney function and hearing loss have been reported. Some evidence indicates that many physiological, pathological and pharmacological similarities exist in the cochlea and kidney [21] and that these similarities may account for the similar effects of some medications and immunological factors on the two organs. The stria vascularis of the cochlea and the glomerulus of the kidney are epithelial structures that are intimately associated with the vascular system [21, 41], and a number of ion channels and transporters involved in K^+^ cycling and endolymphatic K^+^, Na^+^, Ca^2+^, and pH homeostasis are expressed in both the inner ear and kidney [42]. Some studies [43–45] also indicate that abnormalities of nerve conduction in central and peripheral pathways in ESKD patients probably influence the auditory response. Yassin et al. [22] found that the degree of hearing loss is directly related to the degree of hyponatremia, regardless of the level of blood urea, and that cochlear affections are greatly improved by correcting kidney failure and restoring serum Na. Govender et al. [23] reported that cochlear function in patients with CKD could be affected by elevated electrolyte, urea and creatinine levels, concomitant conditions such as hypertension, and ototoxic drugs such as furosemide.

Besides the reduced eGFR, our study also confirmed that health condition factors, including older age, hypertension, diabetes, and stroke, are significantly associated with hearing loss. Hearing loss in older persons may be caused by the death or damage of cochlear sensory hair cells and decreased functioning of the stria vascularis [5]. Explanations for the association between diabetes and hearing loss include microvascular and neuropathic complications affecting diabetics in multiple organ systems that may also affect the inner ear [46]. Duck et al. [47] observed that hypertension and diabetes have a synergistic effect on high-frequency hearing loss. All levels of the auditory pathway, especially the hearing end-organ and auditory nerve, are very vulnerable to stroke damage, which is related to an abnormal arterial blood supply [48]. In contrast to previous studies [49], we did not find an association between obesity, either BMI ≥30 kg/m^2^ or central obesity, and hearing loss. However, overweight (BMI in 25.0–29.9 kg/m^2^) appeared to be a protective factor of hearing loss, which may be due to the low rate of obesity in the middle-aged and elderly Chinese population. Based on the logic of deriving clinical risk scoring system in previous studies [50], the risk of hearing loss would increase when multiple risk factors with OR larger than 1.00 co-exist. This means that a patient with hypertension and diabetes may have higher risk of hearing loss than a patient with only hypertension.

The main strengths of our study include the nationally representative large-scale dataset, high response rate, and strict quality control process. However, certain limitations must also be noted. First, causal inferences could not be drawn based on the current cross-sectional study. Second, information bias may exist as hearing loss was defined on the basis of self-reported results. However, the reliability of self-reported hearing loss has been previously confirmed [28, 29]. For example, Ferrite et al. [29] studied the validity of the following two questions: “Do you feel you have a hearing loss?” and “In general, would you say your hearing is ‘excellent,’ ‘very good,’ ‘good,’ ‘fair,’ ‘poor’?” and revealed that responses to each of the two questions are sufficiently accurate to be used as evidence of hearing loss in epidemiological studies on adult populations. These two questions are similar to the survey questions used in our study. Third, the information about albuminuria, congenital hearing loss, external or middle-ear pathology, use of ototoxic drugs, and exposure to noise, which may be potential confounders in the association between reduced kidney function and hearing loss or causes of hearing loss, was not available in our study. Fourth, we excluded 7058 participants without creatinine data or hearing status, which constituted 36.1% of the total number of participants aged 45 and over in the CHARLS 2015 dataset, and such exclusion may cause selection bias to some extent. The highest data missing rate for some variables including education, area of residence, smoking, drinking, central obesity, hypertension, diabetes, stroke, and LDL cholesterol, was only 3.5%. An unknown category was used to represent missing data for variables with missing rates greater than 1%, and variables with missing rates lower than 1% were not preprocessed as cases containing missing data were automatically deleted during logistic regression analysis. Missing data in these variables may cause some, albeit very limited, selection bias. Finally, the possibility of residual confounding exists.

## Conclusions

This study indicated that reduced kidney function is independently associated with hearing loss in the middle-aged and elderly Chinese and that patients with reduced kidney function have higher odds of hearing loss. We recommend that testing for hearing should be included in the integrated management among patients with CKD, and patients with CKD need to pay attention to their hearing status from early stage to enable the implementation of suitable interventions at an appropriate time and prevent the development of hearing loss.

## Supplementary information

**Additional file 1: Table S1.** Multivariate Logistic regression analysis of hearing loss

## Data Availability

The data that support the findings of this study are available from the CHARLS project team but restrictions apply to the availability of these data, which were used under license for the current study, and so are not publicly available. Data are however available from the authors upon reasonable request and with permission of the CHARLS project team.
