# Supplementary material for: The association between reduced kidney function and hearing loss: a cross-sectional study
Source: BMC Nephrol. 2020 Apr 22;21:145. doi: 10.1186/s12882-020-01810-z (PMC7178984; doi:10.1186/s12882-020-01810-z)
Supplement: Supplementary file 1 — Additional file 1: Table S1. Multivariate Logistic regression analysis of hearing loss [file 12882_2020_1810_MOESM1_ESM.docx]

**Table S1. Multivariate Logistic regression analysis of hearing loss**

| **Variables** | **OR** | **95%CI** | ***p*-value** |
| --- | --- | --- | --- |
| **eGFR (mL/min/1.73 m^2^)** |  |  |  |
| ≥90 | Reference |  |  |
| 60–89 | 1.11 | 1.00-1.23 | 0.043 |
| <60 | 1.25 | 1.04-1.49 | 0.017 |
| **Age (years)** |  |  |  |
| 45–54 | Reference |  |  |
| 55–64 | 1.33 | 1.18-1.51 | <0.001 |
| ≥65 | 2.60 | 2.28-2.97 | <0.001 |
| **Female** | 0.93 | 0.80-1.07 | 0.299 |
| **Education** |  |  |  |
| Illiterate | Reference |  |  |
| Literate | 0.71 | 0.60-0.84 | <0.001 |
| Primary | 0.70 | 0.63-0.78 | <0.001 |
| Middle | 0.56 | 0.49-0.64 | <0.001 |
| High and above | 0.37 | 0.31-0.45 | <0.001 |
| **Rural area** | 1.23 | 1.08-1.40 | 0.001 |
| **Smoking** |  |  |  |
| Never | Reference |  |  |
| Current | 0.96 | 0.84-1.10 | 0.547 |
| Past | 1.23 | 1.05-1.43 | 0.010 |
| **Drinking** |  |  |  |
| Never | Reference |  |  |
| Current | 0.90 | 0.80-1.00 | 0.058 |
| Past | 1.10 | 0.96-1.27 | 0.174 |
| **BMI (kg/m^2^)** |  |  |  |
| <18.5 | 1.18 | 0.99-1.41 | 0.062 |
| 18.5–24.9 | Reference |  |  |
| 25.0–29.9 | 0.86 | 0.76-0.96 | 0.010 |
| ≥30.0 | 1.03 | 0.83-1.29 | 0.779 |
| **Central Obesity** | 0.93 | 0.81-1.06 | 0.263 |
| **Hypertension** | 1.23 | 1.12-1.35 | <0.001 |
| **Diabetes** | 1.23 | 1.10-1.38 | <0.001 |
| **Stroke** | 1.38 | 1.08-1.77 | 0.010 |
| **HDL Cholesterol** | 1.00 | 1.00-1.00 | 0.939 |
| **LDL Cholesterol** | 1.00 | 1.00-1.00 | 0.014 |

Abbreviations: *OR* odds ratio, *CI* confidence interval, *eGFR* estimated glomerular filtration rate, *BMI* body mass index, *HDL* high-density lipoprotein, *LDL* low-density lipoprotein.
